# Soft Palate and Pharyngeal Surgery for the Treatment of Snoring: A Systematic Review

**DOI:** 10.3390/jcm14144964

**Published:** 2025-07-14

**Authors:** Giovanni Cammaroto, Giuseppe Caccamo, Tommaso Rodella, Diletta Angeletti, Francesca Boscolo Nata, Davide Topazio, Luca Cerritelli

**Affiliations:** 1 ENT Unit, Morgagni Pierantoni Hospital, 47121 Forli, Italy; 2 Young Otolaryngologists of the Italian Society of Otolaryngology (GOS-SIO), Italy; giuseppe.caccamo87@icloud.com (G.C.); dilettaangeletti@gmail.com (D.A.); francesca.boscolonata@gmail.com (F.B.N.); davide.topazio@libero.it (D.T.); luca.cerritelli.bo@gmail.com (L.C.); 3 ENT Unit, Santa Chiara Hospital, 56126 Trento, Italy; 4 ENT Unit, University of Ferrara, 44124 Ferrara, Italy; tommaso.rodella13@gmail.com; 5 ENT Unit, Policlinico Umberto I, 00161 Rome, Italy; 6 ENT Unit, Azienda Universitaria Giuliano Isontino, 34128 Trieste, Italy; 7 ENT Unit, Mazzini Hospital, 64100 Teramo, Italy

**Keywords:** snoring, OSAS, surgery

## Abstract

**Background**: Snoring is a common symptom within the spectrum of sleep-disordered breathing, often occurring independently or in association with obstructive sleep apnea syndrome (OSAS). Despite its prevalence, treatment strategies remain variable and lack standardization, particularly regarding surgical interventions. This review aims to evaluate and summarize the outcomes of soft palate and pharyngeal surgeries for adult snoring based on recent literature. **Methods**: A systematic review was conducted using the PubMed database, identifying studies published between 2014 and 2024 that involved adult patients undergoing upper airway surgery for snoring. Inclusion criteria required pre- and postoperative snoring assessment using the Visual Analog Scale (VAS). Studies were categorized by surgical technique (anterior vs. lateral/circumferential), anesthesia type, presence of tonsillectomy, BMI, OSAS severity (based on AHI), and use of Drug-Induced Sleep Endoscopy (DISE). Descriptive analysis was performed on the changes in VAS scores. **Results**: A total of 43 studies involving 2713 patients were included, with 18 eligible for quantitative analysis (716 patients). Across all patients, mean VAS scores improved from 7.29 to 3.50 (ΔVAS 3.79). Both anterior and lateral/circumferential techniques yielded significant symptom reduction (ΔVAS 4.12 and 3.68, respectively). General anesthesia showed slightly better outcomes than local anesthesia. Notably, tonsillectomy was associated with greater symptom improvement (ΔVAS 5.17 vs. 4.49). Patients with lower BMI and milder OSAS showed higher baseline VAS but similar improvements. Limited objective measures and heterogeneity in surgical protocols were key limitations. **Conclusions**: Surgical interventions for snoring provide subjective symptom relief regardless of surgical approach or OSAS severity. Tonsillectomy may enhance outcomes. Future efforts should prioritize standardized, objective outcome measures and personalized treatment planning, potentially incorporating DISE and wearable acoustic technologies.

## 1. Introduction

Snoring represents a cross-sectional entity within the spectrum of sleep-related breathing disorders. It is a condition associated with inspiratory or sometimes expiratory vibration affecting various structures along the airway, and it correlates, in varying percentages, with sleep respiratory disorders. Snoring can be present in upper airway resistance syndrome (UARS), where increased resistance can be documented via flow tracing and arousals identified by EEG or autonomic modification of arterial tonometry, in the absence of apneic or hypopneic events. Furthermore, snoring can be variably associated with obstructive sleep apnea syndrome (OSAS) of any degree (mild, moderate, or severe), where it is linked to the presence of obstructive apneic or hypopneic events. Simple snoring, on the other hand, is a condition with no association to other respiratory disorders.

A multitude of different surgical interventions have been proposed for the treatment of snoring, addressing various anatomical structures (nose, palate, uvula, lateral pharyngeal walls, tongue base, epiglottis), techniques (cold surgeries, laser, injection), and anesthetic approaches (local or general anesthesia). Currently, there is no standard surgical treatment for snoring [1] similar to that for OSAS, and the approach is tailored to the patient′s characteristics. From this perspective, preoperative assessment of the vibratile site is highly recommended. This evaluation could be performed with the patient awake [2] or using drug-induced sleep endoscopy (DISE). While DISE is strongly recommended for OSA surgery, it is not widely performed during preoperative snoring work-ups nowadays [3].

This tailored approach, however, complicates the analysis of surgical outcomes for snoring; the literature mainly consists of pilot studies describing new techniques, while randomized controlled trials comparing different techniques are lacking.

Outcome analysis is also challenging due to the absence of a widely accepted objective measure to evaluate and compare outcomes across studies.

Snoring is commonly evaluated using a combination of subjective and semi-objective questionnaires that assess both the severity of symptoms and their impact on quality of life: the Visual Analog Scale (VAS), NOSE Score (Nasal Obstruction Symptom Evaluation), SF-36, and Snore-25 Questionnaire.

Conversely, objective measures of snoring intensity in terms of loudness expressed in decibels (dB) are less frequent. Notably, there is no universally recognized index for characterizing the magnitude and duration of snoring, unlike the apnea-hypopnea index utilized for OSAS, which is expressed per hour of sleep.

Usually, polysomnographic devices record the percentage of snoring over total sleep time, but the validity of these measurements is still debated.

This work presents a literature review aimed at evaluating and summarizing existing studies over the past decade on adult patients affected by snoring treated with several soft palate and pharyngeal techniques, and their outcomes.

## 2. Materials and Methods

### 2.1. Study Design and Search Strategy

We conducted a literature review to evaluate the effects of pharyngeal and soft palate surgery on snoring severity. The search was performed on the PubMed database using the keywords “snoring” and “surgery”, initially yielding 3261 results. The search was then restricted to studies published within the last 10 years (2014–2024), narrowing the results to 1507.

Further exclusion criteria were applied as follows:

- Non-English articles (n = 100)
- Studies involving pediatric populations (n = 435)
- Systematic reviews or meta-analyses (n = 200)
- Articles not including the word “snoring” in the title
- Duplicate records

**Figure 1:**
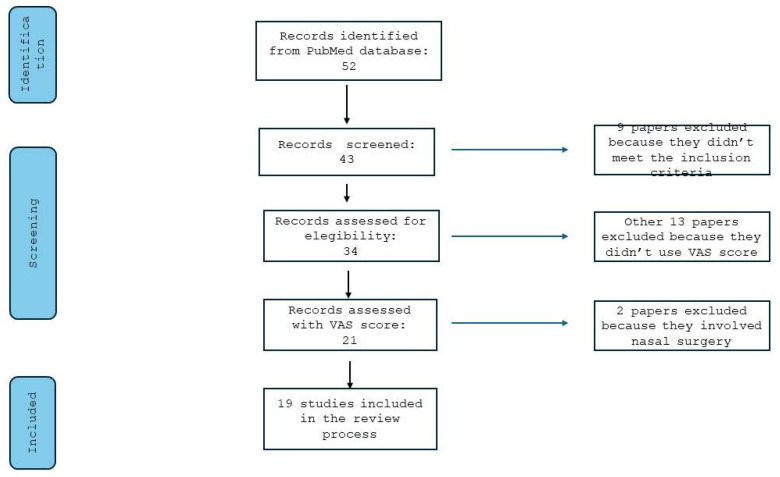
PRISMA flowchart.

After applying all eligibility criteria, a total of 43 studies, selected independently by two reviewers, were included in the qualitative synthesis, involving a total of 2713 patients. The PRISMA flow diagram summarizes the selection process and exclusion rationale with detailed numbers (Figure 1).

### 2.2. Inclusion and Exclusion Criteria

We included clinical studies involving adult patients undergoing upper airway surgery for snoring, with or without obstructive sleep apnea syndrome (OSAS). Eligible studies had to report preoperative and postoperative snoring assessments using the Visual Analog Scale (VAS).

We excluded studies focused on:

- Pediatric populations
- Isolated nasal procedures without snoring-specific outcomes
- Non-surgical or purely pharmacological interventions
- Lack of VAS score
- Case reports

### 2.3. Study Categorization and Surgical Techniques

The selected studies were further categorized based on the following:

1. Type of anesthesia: Patients were grouped according to whether the procedure was performed under general or local anesthesia, as documented in the operative reports.
2. Tonsillectomy: The cohort was divided into two subgroups based on whether or not tonsillectomy was performed in conjunction with the primary surgical procedure.
3. Surgical technique: The surgical approach was classified into two main types as follows:
  - Anterior techniques, which primarily addressed the soft palate and/or uvula, including procedures such as anterior palatoplasties, infiltrative radiofrequency of the soft palate, and palatal implants.
  - Lateral/circumferential techniques, which targeted the lateral pharyngeal wall and/or involved circumferential modifications of the pharyngeal airway. This group included techniques such as lateral pharyngoplasty, expansion sphincter pharyngoplasty, and modified versions of barbed reposition pharyngoplasty with lateral traction (modified BRP).

The classification was based on the main vector of surgical action (anterior vs. lateral/circumferential) as observed during operative planning and intraoperative evaluation. This distinction was employed to evaluate the impact of site-specific surgical approaches on snoring outcomes.

4. Body Mass Index (BMI): patients were stratified into two groups according to their BMI:
  ○ BMI ≤ 27
  ○ BMI > 27
5. Apnea-Hypopnea Index (AHI): patients were categorized based on the severity of preoperative obstructive sleep apnea, using the following thresholds:
  ○ AHI ≤ 30 events/hour
  ○ AHI > 30 events/hour

### 2.4. Preoperative Evaluation and DISE Use

Preoperative evaluation varied among studies. Notably, seven articles reported the use of Drug-Induced Sleep Endoscopy (DISE) to guide surgical planning, while 12 studies did not utilize DISE in the preoperative workup.

### 2.5. Outcome Measure: Visual Analog Scale (VAS)

The Visual Analog Scale (VAS) was used in all included studies as the primary outcome to assess snoring severity, as perceived by either the patient or their bed partner. The scale ranges from 0 (no snoring) to 10 (extremely loud or disruptive snoring). Although subjective, the VAS is widely used in clinical practice due to its simplicity and ability to track symptomatic improvement postoperatively.

For each study, we extracted and compared preoperative and postoperative VAS scores, calculating the difference (ΔVAS) as the primary measure of surgical efficacy.

### 2.6. Data Extraction and Statistical Analysis

Data were independently extracted by two reviewers. The following variables were collected: number of patients, type of surgical procedure, anesthesia type, VAS scores (pre- and post-operative), AHI values, BMI categories, and use of DISE.

A descriptive statistical analysis was conducted to evaluate trends and mean ΔVAS (preoperative-postoperative VAS) within each subgroup. Results were compared across techniques, patient characteristics, and surgical modalities to assess potential factors influencing snoring outcomes.

### 2.7. Risk of Bias Assessment

The risk of bias of the included non-randomized studies was assessed by two reviewers independently using the ROBINS-I (Risk Of Bias In Non-randomized Studies-of Interventions) tool. This instrument evaluates bias across seven domains, including confounding, selection of participants, classification of interventions, deviations from intended interventions, missing data, measurement of outcomes, and selection of reported results.

Due to the observational nature of most included studies, a preliminary judgment of risk of bias was made based on study design and methodology as described in the articles. Each study was categorized as having Low, Moderate, Serious, or Critical risk of bias. Randomized controlled trials were assessed using the same tool for consistency.

## 3. Results

### 3.1. Overall Outcomes

The main patient characteristics collected from the included studies are shown in Table 1.

**Figure 2:**
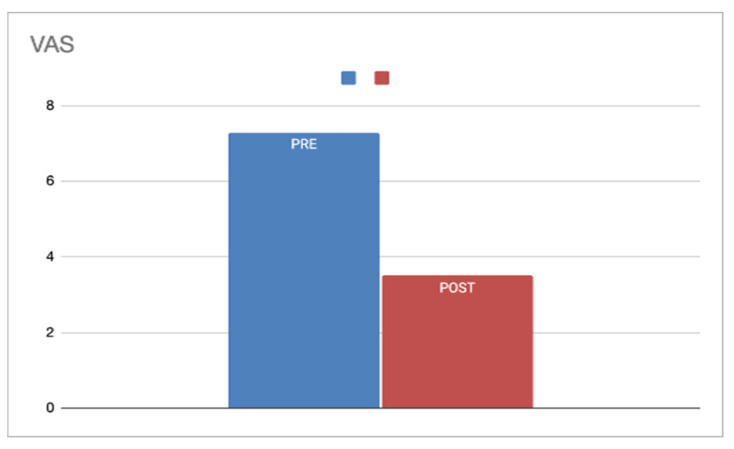
Preoperative (blue) and postoperative (red) mean VAS score.

Across all patients, the mean preoperative VAS score was 7.29, which decreased to 3.50 postoperatively, resulting in an overall ΔVAS of 3.79, indicating a significant subjective improvement in snoring following soft palate and pharyngeal surgery, as shown in Figure 2.

#### 3.1.1. Surgical Technique Comparison

A total of 17 studies out of the 19 included in this review, comprising 716 patients, were eligible for quantitative analysis of snoring severity using the Visual Analog Scale (VAS) pre- and post-operatively. Woodson et al.’s study was eliminated due to lack of data [11], whereas Karakoc et al.’s paper [12] was eliminated due to intervention heterogenicity. Babademez’s study [16] considered two groups of individuals.

The surgical techniques analyzed were divided into two major groups: anterior pharyngoplasty (ANT), aimed to stiffen/reduce soft palate and/or uvula, and lateral/circular pharyngoplasty (LAT/CIRC), focused on stabilization of lateral pharyngeal wall with or without a correction of soft palate flaccidity.

Among the 716 patients, 382 underwent ANT pharyngoplasty, while 334 underwent LAT/CIRC procedures (Table 2).

- In the ANT group, the mean preoperative VAS was 8.11 and decreased to 3.99 postoperatively, resulting in a ΔVAS of 4.12 (Table 3).
- In the LAT/CIRC group, the mean preoperative VAS was lower, at 6.35, with a postoperative VAS of 2.67 and a ΔVAS of 3.68 (Table 3).

**Figure 3:**
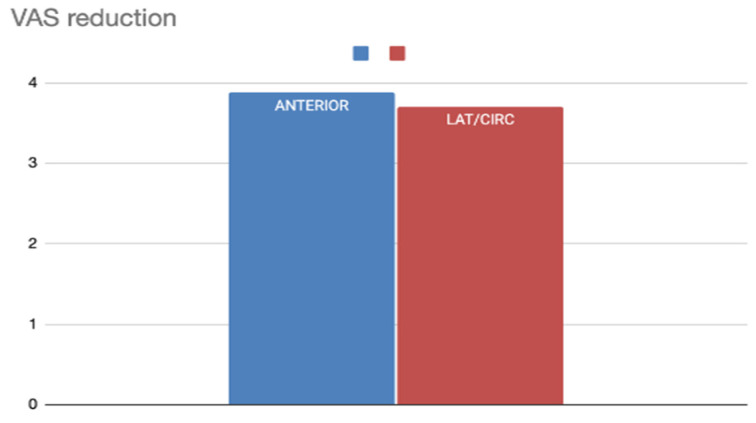
VAS reduction according to the vector of the surgical technique.

Although both techniques resulted in a substantial reduction in snoring, the ANT group showed a slightly higher ΔVAS, despite having a higher baseline VAS score (Figure 3).

#### 3.1.2. Anesthesia Type

The impact of anesthesia type was also considered (Table 3):

- General anesthesia (417 patients): VAS decreased from 6.82 to 2.86 (ΔVAS 3.96).
- Local anesthesia (282 patients): VAS decreased from 8.04 to 4.18 (ΔVAS 3.86).

**Figure 4:**
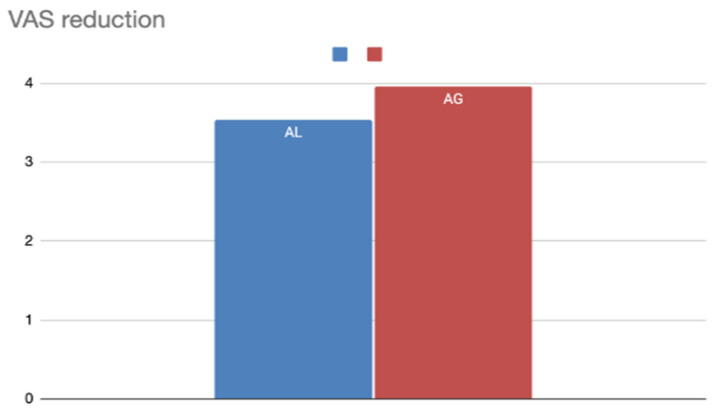
VAS reduction according to the type of anesthesia used.

Both anesthesia types were associated with meaningful improvement, with slightly better outcomes observed in the general anesthesia subgroup (Figure 4).

#### 3.1.3. Tonsillectomy

We also compared patients who underwent tonsillectomy (137 patients) with those who did not (562 patients) (Table 3).

- The tonsillectomy group showed a higher preoperative VAS (7.9), but also a significantly lower postoperative VAS (2.73), resulting in a ΔVAS of 5.17.
- The non-tonsillectomy group had a preoperative VAS of 7.17 and a postoperative VAS of 2.68, with a ΔVAS of 4.49.

**Figure 5:**
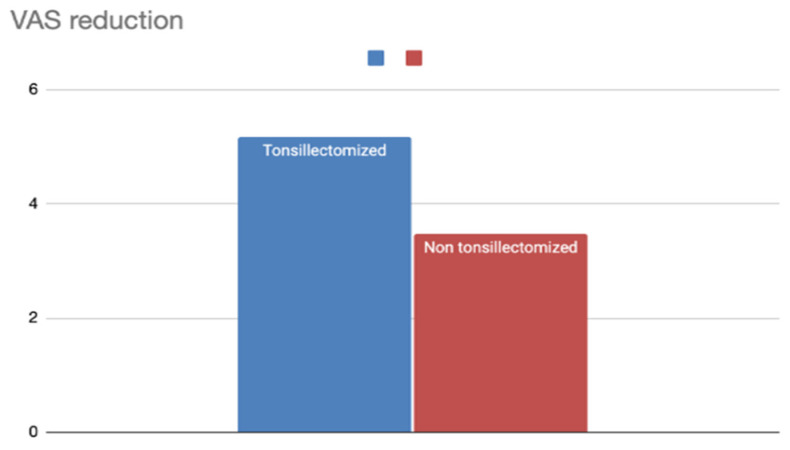
VAS reduction according to the addition of tonsillectomy in the surgical procedure.

These data suggest that the addition of tonsillectomy, when possible, may enhance the efficacy of pharyngeal surgery in reducing snoring severity (Figure 5).

#### 3.1.4. OSAS Severity (AHI)

Apnea-Hypopnea Index (AHI) values were available in 16 studies. Patients were categorized into two groups based on AHI (Table 3):

- AHI ≤ 30 (360 patients): VAS decreased from 8.26 to 4.24 (ΔVAS 4.03).
- AHI > 30 (356 patients): VAS decreased from 6.31 to 2.76 (ΔVAS 3.55).

**Figure 6:**
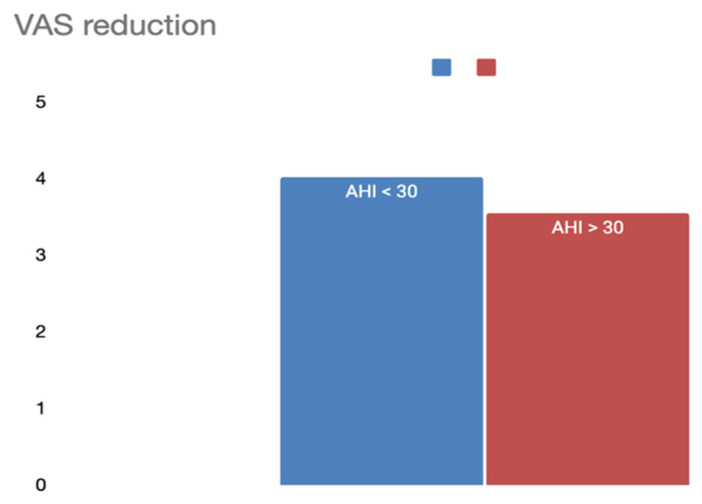
VAS reduction according to preoperative AHI.

The results indicate that while patients with milder OSAS (AHI < 30) had higher baseline VAS scores, both groups experienced a clinically relevant improvement after surgery, with no major difference in ΔVAS between severity groups (Figure 6).

#### 3.1.5. BMI Subgroups

Body Mass Index (BMI) data were analyzed across two categories (Table 3):

- BMI ≤ 27 (253 patients, six studies): VAS decreased from 8.1 to 3.9 (ΔVAS 4.2).
- BMI > 27 (411 patients, 11 studies): VAS decreased from 6.72 to 3.0 (ΔVAS 3.7).

**Figure 7:**
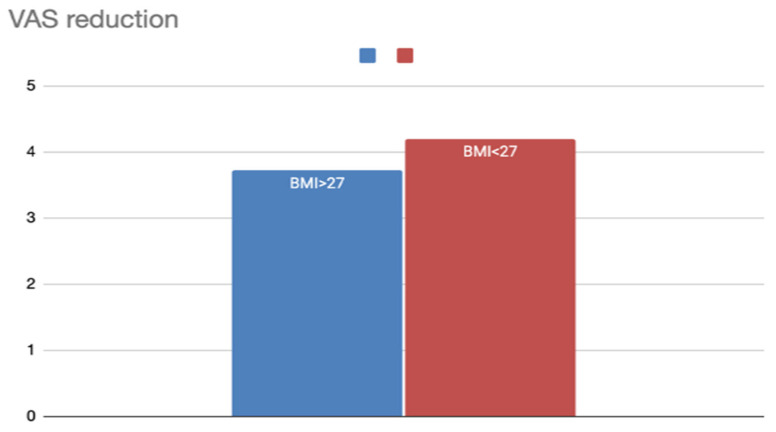
VAS reduction according to preoperative BMI.

Although both groups improved, patients with a lower BMI demonstrated a slightly greater ΔVAS, possibly reflecting better tissue dynamics or reduced anatomical resistance to airflow during sleep (Figure 7).

#### 3.1.6. Risk of Bias Analysis

Of the 19 included studies, most were judged to have a serious or critical risk of bias. Specifically, eight studies were classified as having a serious risk of bias, nine were classified as having a critical risk of bias, and two were judged as moderate risk of bias. Only one study was a randomized controlled trial, and it was assessed as having moderate risk due to potential issues in reporting and follow-up completeness. The predominant reasons for elevated risk were the lack of control groups, the absence of blinding, the retrospective design, and the incomplete adjustment for confounding variables.

## 4. Discussion

This study investigated the efficacy of soft palate and pharyngeal wall surgical procedures in the treatment of snoring, with a main focus on subjective outcomes measured utilizing the VAS score. Included studies seem to show that the choice of surgical technique—targeting either the soft palate or the lateral pharyngeal wall—may not have an impact on subjective snoring changes. Additionally, the severity of OSAS, already known to be associated with the level of upper airway collapsability [23,24], did not appear to be associated with significant changes in the subjective perception (VAS) of snoring severity following surgery. Conversely, included studies reported higher improvements in snoring in patients who underwent tonsillectomy in addition to other pharyngeal and soft palate procedures, in line with the evidence supporting this intervention as an effective procedure for the treatment of sleep-disordered breathing diseases [25].

These findings contribute to the evolving understanding of snoring as a distinct clinical entity, often coexisting with OSAS but not always responding to the same therapeutic strategies. While OSAS management is guided by clearly defined diagnostic and treatment protocols, snoring is frequently addressed based on subjective complaints (VAS score), with treatment efficacy often evaluated without standardized, objective metrics. Furthermore, the lack of significant differences between surgical approaches in our review suggests that other factors—such as tissue vibration characteristics, anatomical variability, and airflow dynamics—may play a more prominent role in the genesis and persistence of snoring than previously recognized.

In this context, the potential benefit of tonsillectomy is noteworthy. Although the size of tonsils and their position are more commonly considered in the context of airway obstruction in OSAS, it is possible that removing the tonsils may reduce soft tissue vibration and the resonant structures that contribute to snoring. This finding aligns with other previous studies suggesting that multilevel surgical interventions, especially those including tonsillar tissue, can lead to greater symptom relief in selected patients. The recent evidence from Moffa et al. (2023) [26] supports this perspective, demonstrating that barbed pharyngoplasty (BP)—a low-invasive technique often employed as part of multilevel surgical procedures—can significantly improve snoring symptoms with a favorable safety profile. Their systematic review highlights that, in appropriately selected patients, barbed pharyngoplasty offers meaningful symptom reduction, and outcomes appear more favorable when the procedure involves treating various anatomical levels, including the tonsillar region. This strengthens the importance of comprehensive airway assessment and individualized surgical planning (Moffa et al., 2023 [26]).

There is a broader trend towards targeting the lateral pharyngeal wall (LPW) as a crucial anatomical structure in snoring and sleep-disordered breathing. In their review, Cammaroto et al. (2020) [27] show the growing interest in surgical techniques that manipulate the LPW musculature, such as lateral pharyngoplasty and barbed reposition pharyngoplasty, due to their potential to reduce upper airway collapsibility and tissue vibration. Even if our study did not demonstrate significant differences in subjective snoring outcomes between procedures targeting the soft palate versus the LPW, the literature suggests that careful identification of LPW-related collapse may improve patient selection and outcomes. Furthermore, the low complication rates reported by Cammaroto et al. across LPW-targeting procedures support their role as safe options within a multilevel surgical framework. This highlights the need to further refine phenotyping of airway obstruction patterns to optimize surgical targeting (Cammaroto et al., 2020 [27]).

Despite these findings, several important limitations must be addressed.

The overall quality of the evidence included in this review is limited by the risk of bias inherent in non-randomized studies. While the findings offer valuable insights into surgical approaches for snoring, the lack of control groups and prospective randomization in most studies increases the potential for confounding and selection bias. These limitations should be considered when interpreting the results and underscore the need for well-designed randomized trials to strengthen the evidence base in this field.

One of the most prominent limitations is the heterogeneity of the surgical techniques. Although this reflects normal clinical practice, where usually the procedures are tailored to individual anatomical, functional characteristics and physicians’ preferences, it introduces a variability that limits the generalizability of our conclusions. Additionally, the study relied only on subjective assessments of snoring using the VAS, without including important objective measures, such as acoustic analysis or audio recordings during polysomnography. In particular, the lack of quantitative acoustic analysis—including various parameters such as pitch, intensity, and frequency variability of snoring—represents a big gap, since these characteristics could be crucial for a comprehensive and reproducible evaluation of snoring severity and its consequent social impact. Recently, some authors developed a new technology based on acoustic signal evaluation aimed at diagnosing OSAS [28]; these efforts highlight the need for caregivers to find effective and easy alternatives to conventional diagnostic devices, in order to offer reliable diagnoses and easily repeatable recordings in patients affected by sleep-disordered breathing diseases. In this sense, the adoption of wearables appears to be an interesting solution, capable of continuously collecting acoustic data that may help in the standardization of snoring diagnosis and staging. Since some devices already available on the market have recently been FDA-approved for the detection of signs of moderate to severe OSAS, and several mobile apps for recording snoring have been designed, further effective steps toward precise phenotyping of snoring seem close to being taken [29].

Subjective measures, while they are valuable for recording patient-centered outcomes, are, nevertheless, evidently susceptible to bias and may not accurately reflect the physiological characteristics of the snoring event.

Additionally, the majority of studies only report on mid-term follow-up (6–12 months), which does not provide a perspective on the stability of the results over time.

Another significant limitation lies in the limited exploration of nose surgery as part of the overall treatment plan for snoring. While nasal obstruction is a frequent and well-documented contributor to increased upper airway resistance and turbulent airflow, which can exacerbate snoring, our data did not permit a focused analysis on the impact of nasal procedures. If we consider the high prevalence of nasal obstruction in the overall population and its potential role in promoting mouth breathing and palatal flutter, it is important that future studies should aim to better redefine the role of nose surgery in the overall management of snoring. Recent studies have shown that nasal surgery, particularly in patients with nasal obstruction, may offer a valuable adjunctive approach to improving snoring. Several investigations have demonstrated significant reductions in snoring intensity and frequency following nasal procedures. For example, Li et al. (2008) reported that 86% of patients experienced improvement in snoring after septoplasty, with 12% achieving complete resolution [30]. Similarly, Taziki et al. (2014) observed significant reductions in Snore Symptom Inventory (SSI) and Epworth Sleepiness Scale (ESS) scores following septoplasty in patients with a deviated nasal septum and habitual snoring [31]. Long-term benefits have also been reported by Walker et al. (2020), who found that 625 patients undergoing functional nasal surgery showed improved Snore Outcome Survey (SOS) scores up to 24 months postoperatively [32].

Another challenge in the field remains the lack of standardized, reproducible methods for objectively evaluating snoring. Currently, there are no established objective criteria that can accurately evaluate the effectiveness of surgical treatment for snoring. The assessment of treatment outcomes largely depends on subjective reports, often provided by the patient or their partner, and lacks the rigor of scientifically validated measures. Unlike other conditions with well-defined, objective diagnostic tools, snoring treatment is often assessed based on anecdotal or subjective feedback, making it difficult to reach universally shared, reproducible, and objective conclusions. Recent evidence, including that of Moffa et al. (2023) [26], underscores the importance of using multilevel interventions that target multiple anatomical levels. Their findings suggest that such approaches provide significant symptom relief for snoring, emphasizing the necessity of an overall comprehensive airway evaluation [26,27,33].

The role of Drug-Induced Sleep Endoscopy (DISE) in the management of obstructive sleep apnea (OSA) has been increasingly supported by the literature, with growing evidence suggesting that pre-operative DISE can significantly influence surgical planning and improve treatment outcomes. Green et al. (2019) demonstrated that DISE findings, particularly those involving lateral pharyngeal wall and tongue base collapse, were associated with less favorable surgical outcomes, indicating the importance of individualized surgical strategies guided by DISE findings [34]. Similarly, in a randomized controlled trial by Iannella et al. (2022), patients undergoing barbed pharyngoplasty with preoperative DISE had a significantly higher therapeutic success rate (83%) compared to those without DISE evaluation (60%) [35]. These findings underscore the potential benefits of incorporating DISE into surgical planning for OSA patients, as it enables a more tailored approach, enhancing the likelihood of a favorable outcome.

Interestingly, the question arises of whether this diagnostic tool can also be applied to primary snoring, a condition often viewed as a less severe manifestation of the same upper airway collapsibility seen in OSA. Although limited evidence directly addresses the impact of DISE on snoring outcomes, its ability to dynamically identify the specific anatomical sites of vibration and obstruction may allow for more targeted interventions, potentially increasing the success rate of procedures such as uvulopalatopharyngoplasty (UPPP), radiofrequency ablation, or minimally invasive palatal implants. The findings from Iannella et al. (2022) support this hypothesis, demonstrating that the use of DISE to guide barbed reposition pharyngoplasty (BRP) in OSA patients significantly improves therapeutic success (83% vs. 60% in the non-DISE group) by identifying specific collapse sites and refining surgical indications [35]. This tailored approach may also benefit patients with isolated snoring, allowing for more effective treatments that target the relevant obstructed areas.

Additionally, wearable technologies are emerging as a potential solution to address this problem. These devices offer the possibility of non-invasive, cost-effective, and long-term monitoring of sleep-related sound pitch and parameters in a home setting. Unlike single-night laboratory recordings, wearables can capture inter-night variability and provide more ecologically valid data across multiple sleep cycles. This could greatly enhance our ability to assess treatment outcomes, stratify patients, and personalize therapeutic strategies. The integration of wearable-based phenotyping with DISE findings may further refine surgical indications and facilitate outcome prediction models in both OSA and primary snoring populations.

In this context, studies by Zhang et al. (2023) and Liu et al. (2022) have emphasized that while DISE may not uniformly improve all surgical outcomes in OSA patients, it facilitates a more tailored surgical approach, which could also benefit patients with isolated snoring [36,37]. Given the shared anatomical and functional characteristics of snoring and OSA, it is plausible that DISE-guided surgery, supported by continuous home-based monitoring, could reduce treatment failure by minimizing unnecessary or misdirected procedures.

Future studies should, therefore, focus on evaluating the effectiveness of DISE in improving surgical outcomes specifically for primary snoring. Randomized controlled trials comparing DISE-guided versus standard assessment in snoring patients—potentially complemented by wearable monitoring—could provide further evidence for incorporating these tools into routine preoperative planning.

## 5. Conclusions

In conclusion, our study suggests that neither the choice of palatal versus lateral pharyngeal surgical approach nor the severity of OSAS seems to influence subjective post-operative snoring outcomes. Furthermore, the integration of tonsil surgery, when possible, may enhance the effectiveness of these interventions. Finally, the adoption of objective measurements appears to be necessary in order to better phenotype snoring patients and possibly adopt tailored individualized therapeutic approaches that may lead to more effective results.

## Figures and Tables

**Table 1:** Characteristics of the included studies (BRP, barbed repositioning pharyngoplasty, MBRP, modified barbed repositioning pharyngoplasty).

	Title	Author (Year)	Study Design	Level of Evidence	Number of Patients	Mean Age	Snoring/OSA	DISE	Mean Follow-Up
1	A new office-based procedure for treatment of snoring: The S.I.Le.N.C.E. study	Friedman M et al. (2020) [4]	Prospective	Level 4	52	n.s.	Snoring	No	6 months
2	Barbed snore surgery for concentric collapse at the velum: The Alianza technique	Mantovani M et al. (2017) [5]	Pilot Longitudinal Study	Level 4	19	43.8	Mild-moderate OSA	Yes	6 months
3	The effectiveness of combined tonsillectomy and anterior palatoplasty in the treatment of snoring and obstructive sleep apnea (OSA)	Adzrel B et al. (2016) [6]	Prospective	Level 4	31	35	Snoring +/− OSA	No	74 months
4	An absorbable thread suture technique to treat snoring	Kwon JW et al. (2015) [7]	Observational	Level 4	35	34.2	Snoring, mild OSA	No	90 days
5	Changes of snoring sound after relocation pharyngoplasty for obstructive sleep apnea: the surgery reduces mean intensity in snoring which correlates well with apoea-hypopnea index	Li HY et al. (2015) [8]	Prospective Case Series	Level 4	32	39	OSA	No	6 months
6	Optimal application of soft-palate webbing flap pharyngoplasty combined with nasal surgery for surgical treatment of primary snoring and obstructive sleep apnea.	Park JA et al. (2022) [9]	Retrospective	Level 3	174	45.1	OSA	Yes	6 months
7	The Efficacy of Diode Laser Palatoplasty on Patients with Troublesome Snoring	Dawood MR et al. (2020) [10]	Prospective Interventional	Level 4	46	45.31	Snoring	No	6 months
8	Radiofrequency ablation of the lateral palatal space for snoring	Tucker Woodson B et al. (2018) [11]	Retrospective Chart Review	Level 3	20	n.s.	Snoring	No	n.s.
9	A tertiary center experience with velopharyngeal surgical techniques for treatment of snoring and obstructive sleep apnea	Karacoc O et al. (2018) [12]	Prospective Series	Level 4	93	40.7	Snoring or OSA	No	5.90 months
10	The treatment of snoring by radiofrequency-assisted uvulopalatoplasty and results after one-session protocol: a prospective, longitudinal, non-randomized study	Chiesa Estomba CM et al. (2015) [13]	Prospective Longitudinal Non Randomized	Level 4	27	49	Snoring	No	12 months
11	Soft palatal webbing flap palatopharyngoplasty for both soft palatal and oropharyngeal lateral wall collapse in the treatment of snoring and obstructive sleep apnea: a new innovative technique without tonsillectomy	Elbassiouny AMME (2015) [14]	Single-Center Prospective Uncontrolled Case Series	Level 4	28	41.3	Snoring or OSA	Yes	6 months
12	No-cutting remodeling intra-pharyngeal surgery can avoid CPAP in selected OSA patients: Myth or reality?	Casale M. et al. (2022) [15]	Prospective Clinical Trial	Level 4	26	52.7	OSA	Yes	6.5 months
13	Technical update of barbed pharyngoplasty for retropalatal obstruction in obstructive sleep apnea	Babademez MA et al. (2019) [16]	Prospective	Level 2	34	BRP = 39.4 MBRP = 40.1	Mild-moderate OSA	Yes	8 months
14	Modified barbed soft palatal posterior pillar webbing flap palatopharyngoplasty	Elbassiouny AMME (2016) [17]	Prospective Single Center Uncontrolled Case Series	Level 4	21	31.3	OSA	Yes	6 months
15	Barbed anterior pharyngoplasty: An evolution of anterior palatoplasty	Salamanca F et al. (2014) [18]	Not Specified	Level 4	24	46	Snoring or mild OSA	Yes	6 weeks
16	Treatment of snoring using a non-invasive Er:YAG laser with SMOOTH mode (NightLase): a randomized controlled trial.	Picavet VA et al. (2023) [19]	Prospective 20 vs. 20	Level 2	40	43.3 vs. 44.5	Snoring or mold OSA	No	3 months
17	Radiofrequency of the soft palate for sleep-disordered breathing: a 6-year follow-up study	De Kermadec H et al. (2014) [20]	Observational Retrospective	Level 4	77	52.2	Mild-moderate OSA	No	6.3 years
18	Outpatient erbium:YAG (2940 nm) laser treatment for snoring: a prospective study on 40 patients.	Storchi IF et al. (2018) [21]	Prospective	Level 4	40	53.1	Snoring and mild, moderate, severe OSA	No	20 months
19	The palatal septal cartilage implantation for snoring and obstructive sleep apnea	Lee YC et al. (2018) [22]	Retrospective	Level 4	10	45	Snoring and OSA	No	12 months

**Table 2:** VAS values according to the vector of surgical technique.

Authors	References	Year	N	Technique	Type	VAS Pre (Mean ± DS)	VAS Post (Mean ± DS)	VAS (Mean)
Li et al.	[8]	2015	32	Relocation Pharingoplasty	Lat/circ	7.5 ± 1.9	2.6 ± 1.8	4.9
Casale et al.	[15]	2022	26	Alianza	Lat/circ	7.85 ± 1.23	3.2 ± 1.7	4.65
Babademez et al.	[16]	2019	17	Modified barbed pharyngoplasty	Lat/circ	8 ± 1.5	1.8 ± 0.8	6.2
Babademez et al.	[16]	2019	17	Barbed pharyngoplasty	Lat/circ	6.2 ± 1.9	2.2 ± 1	4
Elbassiouny et al.	[17]	2016	21	Modified barbed pharyngoplasty	Lat/circ	9.4 ± 1.6	1.7 ± 3.2	7.7
Bakri Adzreil et al.	[6]	2016	31	Anterior pharyngoplasty and tonsillectomy	Ant	7.3 ± 1.0	2.9 ± 1.6	4.4
Lee et al.	[22]	2018	10	Septum Cartilage Implantation	Ant	8 ±0.74	4.5 ± 2.59	3.5
Friedman et al.	[4]	2020	52	Elevoplasty	Ant	7.81 ± 1.9	5.4 ± 2.2	2.41
Mantovani et al.	[5]	2017	19	Alianza	Lat/circ	9.5 ± 0.7	2.1 ±1.7	7.4
Jangwoo Kwon et al.	[7]	2015	35	Stiffness of the soft palate	Ant	8.74 ± 2.28	4.14 ± 1.68	4.6
Park et al.	[9]	2022	174	Soft palatal webbing flap pharyngoplasty and nasal surgery	Lat/circ	4.7	2.9	1.8
Dawood et al.	[10]	2020	46	Diode Laser Palatoplasty	Ant	6.6	2.4	4.2
Estomba et al.	[13]	2015	27	RFA Uvulopalatoplasty	Ant	8.10 ± 0.93	4.90 ± 0.77	3.2
Elbassiouny et al.	[14]	2015	28	Soft palatal webbing flap pharyngoplasty	Lat/circ	8.6 ± 3.7	2.3 ± 2.7	6.3
Salamanca et al.	[18]	2014	24	Barbed anterior pharyngoplasty	Ant	9.2	2.9	6.3
Picavet et al.	[19]	2023	40	Laser palatoplasty	Ant	7.9 ± 2.0	4.7 ± 2.8	3.2
De Kermadec et al.	[20]	2014	77	RFA Uvulopalatoplasty	Ant	8.1 ± 1.5	5.7 ± 2.9	2.4
Storchi et al.	[21]	2018	40	Laser pharyngoplasty	Ant	10 ± 1.48	3 ± 2.96	7

**Table 3:** Studies classification according to surgery and anesthesia characteristics, BMI, AHI and VAS.

	Patients	VAS Pre	VAS Post	ΔVAS	
**Anesthesia**					
general	417	6.82	2.86	3.96	
local	282	8.04	4.18	3.86	
**Tonsillectomy**					
yes	137	7.9	2.73	5.17	
no	562	7.17	2.68	4.49	
**Surgical technique**					
Anterior pharyngoplasty	382	8.11	3.99	4.12	
Lateral/Circular pharyngoplasty	317	6.35	2.67	3.68	
**AHI**					
AHI > 30		356	8.26	4.23	4.03
AHI < 30		360	6.31	2.76	3.55
**BMI**					
BMI < 27		253	8.1	3.9	4.2
BMI > 27		411	6.72	3	3.7

## Data Availability

This systematic review was not registered. Review protocol can be accessed if requested. The datasets generated during and/or analysed during the current study are available from the corresponding author on reasonable request.

## Abbreviations

OSA	obstructive sleep apnea
BRP	barbed repositioning pharyngoplasty
AHI	Apnea Hypopnea Index
BMI	Body mass index
DISE	drug induced sleep endoscopy
VAS	visual analog scale
